# *Mycoplasma pneumoniae* co-infection with SARS-CoV-2: A case report

**DOI:** 10.1099/acmi.0.000212

**Published:** 2021-03-10

**Authors:** Rama Chaudhry, K. Sreenath, E. V. Vinayaraj, Biswajeet Sahoo, M. R. Vishnu Narayanan, K. V. P. Sai Kiran, Priyam Batra, Nisha Rathor, Sheetal Singh, Anant Mohan, Sushma Bhatnagar

**Affiliations:** ^1^​ Department of Microbiology, All India Institute of Medical Sciences, New Delhi, India; ^2^​ Department of Laboratory Oncology, National Cancer Institute, Jhajjar, Hayana, India; ^3^​ Department of Anaesthesiology, All India Institute of Medical Sciences, New Delhi, India; ^4^​ Department of Hospital Administration, National Cancer Institute, Jhajjar, Hayana, India; ^5^​ Department of Pulmonary, Critical Care and Sleep Medicine, All India Institute of Medical Sciences, New Delhi, India; ^6^​ Department of Onco-Anaesthesia and Palliative Medicine, All India Institute of Medical Sciences, New Delhi, India

**Keywords:** COVID-19, *Mycoplasma pneumoniae*, infection, pneumonia, therapy, co-infections

## Abstract

We report co-infection with severe acute respiratory syndrome coronavirus 2 (SARS-CoV-2) and *Mycoplasma pneumoniae* in a patient with pneumonia in India. Atypical bacterial pathogens causing community-acquired pneumonia may share similar clinical presentations and radiographic features with SARS-CoV-2 making a thorough differential diagnosis essential. The co-infection of SARS-CoV-2 and *M. pneumoniae* is infrequently reported in the literature. Broader testing for common respiratory pathogens should be performed in severe COVID-19 cases to rule out other concurrent infections. Early identification of co-existing respiratory pathogens could provide pathogen-directed therapy, and can save patient lives during the ongoing COVID-19 outbreak.

## Introduction

In December 2019, an infection due to a novel coronavirus, later named as SARS-CoV-2, was first identified in Wuhan, Hubei province, PR China [1]. Within a period of 3 months, the outbreak initially presented as unexplained pneumonia became the COVID-19 pandemic affecting more than 230 countries, areas, or territories [2]. As of 28 October 2020, 43.76 million confirmed cases have been registered worldwide with more than 1163 thousand fatalities and the disease course is still evolving, posing a serious threat to public health and the global economy [3]. The majority of the cases are mild and self-limiting, however, severe cases with pneumonia and acute respiratory distress syndrome (ARDS) require hospitalization, intensive care, ventilator support, and may often lead to death, especially in older adults [4]. Co-infections with additional pathogens are a common feature of pandemics [5]. Recently, with the advancement of molecular techniques including multiplex PCR, the co-infections involving the respiratory tract are increasingly being identified in severely ill patients. Lansbury *et al*. have reported bacterial and viral co-infections in 7 and 3 % of hospitalized patients with COVID-19 [6]. The co-infection of SARS-CoV-2 and *Mycoplasma pneumoniae* is infrequently reported in the literature [7, 8]. Here, we report a case of pneumonia having co-infection with SARS-CoV-2 and *M. pneumoniae* in India.

## Case report

On 2 July 2020 an adult patient was admitted to the COVID care facility of the National Cancer Institute (NCI), Jhajjar, Haryana, India (an extension of All India Institute of Medical Sciences [AIIMS], New Delhi) with symptoms of fever, shortness of breath, and dry cough for the past 4 days. The patient’s other medical problems included type 2 diabetes mellitus and systemic hypertension. The patient’s vitals were BP-140/76 mmHg, PR-90/min and respiratory rate-20/min. The patient had oxygen saturation (SPO2) of 93 % in room air. The patient tested positive for SARS-CoV-2 by real-time reverse transcription (RT)-PCR in oropharyngeal and nasopharyngeal swabs.

The initial laboratory reports showed haemoglobin of 12.5 g dl^−1^ (reference range 12–15 g dl^−1^), erythrocyte count 4.29×10^6^µl^−1^ (reference range 3.8–4.8×10^6^ µl^−1^), platelet count 166×10^3^ µl^−1^ (reference range 150–400×10^3^ µl^−1^), leucocyte count 9.63×10^3^ µl^−1^ (reference range 4–11×10^3^ µl^−1^) with neutrophils 85.1 % (reference range 40–80 %), lymphocytes 7.3 % (reference range 20–40 %), eosinophils 0.1 % (reference range 0–7 %), monocytes 5.6 % (reference range 3–11 %) and basophils 0.9 % (reference range 0–2 %). The d-dimer level was 282 ng µl^−1^ (reference <500 ng µl^−1^), and fibrinogen raised to 544 mg dl^−1^ (reference range 180–350 mg dl^−1^). The blood levels of C-reactive protein (CRP) 13.58 mg dl^−1^, IL-6 765.5 pg ml^−1^ (reference range 0–4.4 pg ml^−1^), ferritin 1022.6 ng ml^−1^ (reference range 10–291 ng ml^−1^), and LDH 493 U l^−1^ (reference range 120–246 U l^−1^) were all raised. The initial liver and renal function (LFT and RFT) tests were within normal range. Serum calcium (7.6 mg dl^−1^ [reference range 8.7–10.4 mg dl^−1^]) and phosphorous levels (1.95 mg dl^−1^ [reference range 2.4–5.1 mg dl^−1^]) were lower.

Chest radiography of the patient showed the presence of bilateral patchy fluffy opacities in the lung. The patient received antibiotic therapy, including Cefaperazone-sulbactam (2 g/ twice a day, IV), Targocid (400 mg/day, IV) and Levofloxacin (750 mg/day, IV) and Methylprednisolone (40 mg IV once a day) combined with supportive care. Prophylactic anticoagulation therapy was started with enoxaparin 0.4 ml subcutaneously (twice a day). Glycaemic control was achieved with IV insulin infusion as per the sliding scale.

The patient’s clinical status deteriorated with a declining saturation level on room air. A trial of non-invasive ventilation with continuous positive airway pressure (C-PAP) was attempted. However, the C-PAP trial failed and the patient continued to have low SPO2 levels with clinical evidence of ARDS. The patient was intubated because of worsening of respiratory status with tachypnea and disorientation and subsequently placed on mechanical ventilation with volume assist-control mode (ACV), as per ARDS protocol in an intensive care unit. The patient was haemodynamically stable during the initial course of hospital stay.

Throat swab, urine and blood samples of this patient were received for atypical pneumonia investigation. A *Legionella* urinary antigen test (Alere, BinaxNOW, Scarborough, ME) was negative for *Legionella pneumophila* serogroup 1. Immunoglobulin M (IgM) serology by commercial ELISA kit (Euroimmun, UK) was negative for *M. pneumoniae* and *Chlamydia pneumoniae *. Real-time PCR of the throat swab tested positive for *M. pneumoniae CARDS toxin* gene (cycle threshold value was 34) using previously described primers (Mp181-F TTTGGTAGCTGGTTACGGGAAT; Mp181-R GGTCGGCACGAATTTCATATAAG), probe (Mp181-P 6-FAM-TGTACCAGAGCACCCCAGAAGGGCT-MGB NFQ) and cycling conditions [9]. The patient’s throat swab was further tested for 33 respiratory pathogens including 21 viruses and 12 bacteria using the Fast Track Diagnostics Respiratory Pathogen 33 (FTD Resp-33) kit, (Fast Track Diagnostics, Luxembourg) following the manufacturer’s instructions and reaction conditions. The assay was used with the Bio-Rad CFX96 thermocycler. The assay is based on multiplex one-step RT-PCR with probes for simultaneous detection of 33 respiratory pathogens: influenza A virus (IAV), influenza A (H1N1) virus (swine lineage) (IAV[H1N1] swl), influenza B virus (IBV), influenza C virus (IVC), human coronaviruses (HCoV) NL63, 229E, OC43 and HKU1, human parainfluenza viruses (HPIV) 1, 2, 3 and 4, human metapneumoviruses (HMPV) A and B, human rhinovirus (HRV), human respiratory syncytial viruses (HRSV) A and B, human adenovirus (HAdV), enterovirus (EV), human parechovirus (HPeV), human bocavirus (HBoV), *Pneumocystis jirovecii*, *M. pneumoniae *, *Chlamydophila pneumoniae *, *Streptococcus pneumoniae *, *Haemophilus influenzae B*, *Staphylococcus aureus *, *Moraxella catarrhalis *, *Bordetella* spp., *Klebsiella pneumoniae *, *Legionella pneumophila */* Legionella longbeachae *, *Salmonella* spp., *Haemophilus influenzae* and equine arteritis virus (EAV), which serves as an internal control (IC). The patient tested positive only for *M. pneumoniae* by the FTD Resp-33 kit.

The patient remained on Levofloxacin (750 mg/day, IV) along with other antibiotics. Due to persistently raised inflammatory markers, a single dose of Tocilizumab 800 mg was also given intravenously.

Any attempt of weaning was unsuccessful and the patient was kept on pressure-regulated volume-controlled ventilation (PRVC). Oxygenation was maintained with a fraction of inspired oxygen (FiO2) of 60 %. Due to unsuccessful weaning and prolonged ventilation, tracheostomy was performed after 12 days of hospital stay. The patient also developed sepsis that worsened with ventilator-associated pneumonia (VAP) and showed raised procalcitonin levels for which the antibiotics were upgraded to Colistin 4.5MU, twice a day, IV, and Cefipime-Tazobactam 1 g/12.5 mg, twice a day, IV. The patient also developed acute kidney injury (non-oliguric) with raised creatinine levels. Despite all supportive measures, the patient’s clinical status continued to deteriorate and subsequently developed pneumothorax for which an intercostal drain (ICD) was placed. Besides, the patient’s lung expansion was poor, complicated further by the development of a bronchopleural fistula (BPF). An ECG showed a trifascicular block. Despite the treatment with antibiotics, ventilation and supportive care, the patient passed away after 23 days of hospitalization due to bradycardia followed by cardiac arrest on 25 July 2020.

## Discussion

Co-infections in COVID-19 can be potentially lethal; however, data regarding co-infection or secondary infections are limited and still emerging [10]. Further characterization of bacterial co-infections in COVID-19 can enhance clinical management. The identification of these co-infections can be complex as the patient might have harboured the pathogen before viral infection, the organism might be present as a part of an underlying chronic disease or possibly acquired from the hospital [11]. Patients who are hospitalized for a prolonged time should be sampled throughout the disease course and tested for a wide range of common respiratory pathogens using FDA-approved multiplex PCR panels for the identification of co-infections or mixed infections. Clinicians should adopt empirical antibiotics, covering all possible pathogens in severe COVID-19 cases in which co-infections cannot be ruled out.

Cases of SARS-CoV-2 and *M. pneumoniae* co-infections and secondary infections have been reported in the literature; however, the exact rates of these co-infections remain unknown. Blasco *et al.*, Easom *et al.* and Wu *et al.* have reported co-infections due to *M. pneumoniae* in patients with SARS-CoV-2 infections by using molecular assays. Oliva A *et al*., Zhang *et al.*, Gao *et al.*, Chen *et al.* and Gayam *et al.* have reported co-infections of SARS-CoV-2 with *M. pneumoniae* based on serology [7, 12–18].

The pulmonary symptoms of *M. pneumoniae* pneumonia (MPP) may resemble viral infections ranging from mild, self-limiting illness to more severe presentations with ARDS and multi-organ failure [19]. Both COVID-19 pneumonia and MPP have similar symptoms, including fever, cough and shortness of breath [18]. Besides, the similarities in the chest radiographs and HRCT patterns in MPP and COVID-19 make its differential diagnosis difficult. Therefore, under these circumstances, microbiological identification methods are warranted. Detection of nucleic acid from respiratory samples is a direct evidence for diagnosis of *M. pneumoniae* [20]. Azithromycin remains the drug of choice for *M. pneumoniae* infection; nevertheless, fluoroquinolones and tetracyclines are also useful [21]. Currently, no antiviral treatment is specifically approved for COVID-19 and the management is mainly symptomatic, by supportive care and oxygen therapy. However, treatment with hydroxychloroquine, remdesivir, tocilizumab, lopinavir/ritonavir have been used [18]. As the treatment is completely different, the timely detection of co-infection is essential. Even though we identified co-infection with *M. pneumoniae* in our patient, the outcome was fatal due to concurrent conditions and complications developed during the hospital stay.

To conclude, *M. pneumoniae* and SARS-CoV-2 co-infections might occur in patients with pneumonia. Clinicians should suspect co-infections with typical and atypical bacterial pathogens in patients with COVID-19. Broader testing for common respiratory pathogens should be performed in severe COVID-19 cases to rule out other concurrent infections. Lastly, an early identification of co-existing respiratory pathogens could provide targeted antimicrobial treatment, and can save patient lives during the ongoing COVID-19 outbreak.

### Availability of data and material

All the data generated or analysed during this study are included in this article.
